# A review on the integration of ultrasonication in hybrid systems for enhanced hydrogen yield^☆^

**DOI:** 10.1016/j.ultsonch.2025.107552

**Published:** 2025-09-06

**Authors:** Slimane Merouani, Aissa Dehane, Oualid Hamdaoui

**Affiliations:** a Department of Chemical Engineering, Faculty of Process Engineering, University Salah Boubnider Constantine 3, P.O. Box 72, 25000 Constantine, Algeria; b Department of Process Engineering, Faculty of Process Engineering, University Salah Boubnider Constantine 3, P.O. Box 72, 25000 Constantine, Algeria; c Chemical Engineering Department, College of Engineering, King Saud University, 12372 Riyadh, Saudi Arabia

**Keywords:** Hydrogen, Ultrasonication (US), US/photolysis, US/photocatalysis, US/electrolysis

## Abstract

This review synthesizes recent developments in ultrasonication (US)/assisted and US/hybrid processes for hydrogen generation, with a focus on US/electrochemical techniques. It summarizes recent findings, discusses existing constraints, and suggests promising routes for further advancement. US/hybrid processes, including US/electrocatalytic techniques and other US-assisted methods, show great promise in improving efficiency and reducing the energy needed for hydrogen generation. The paper emphasizes how ultrasonication can accelerate electrochemical processes, improve mass transfer, and reduce overpotentials. Ultrasonication enhances the physical and chemical parameters of US/electrocatalytic processes by decreasing cell voltage and overpotentials while boosting overall energy efficiency. Other ultrasonication hybrid processes, such as sonocatalysis and US/photocatalysis, have demonstrated the potential to use ultrasonication to activate catalysts and increase hydrogen yields. Notwithstanding these progresses, difficulties remain, such as improving the understanding of the mechanisms underlying US-enhanced hydrogen generation; optimizing operating conditions (e.g., frequency, acoustic power, electrode materials, and solution temperature); and studying hydrogen production from non-aqueous solutions. This review provides a comprehensive framework for future investigation in this evolving field.

## 1 Introduction

As a prospective clean technology for a variety of chemical processes, including the generation of hydrogen, sonochemistry, the study of chemical reactions generated or accelerated by ultrasonication (US), has drawn a lot of attention [1]. At the core of sonochemistry is acoustic cavitation, which emerges when high-intensity sonolytic waves pass thru a liquid medium, producing small bubbles that expand and burst violently due to pressure changes [2]. This process generates reactive species (e.g., hydrogen atoms, hydroxyl radicals, and hydrogen peroxide). Acoustic cavitation creates localized extreme conditions, including high temperatures (several thousand degrees Celsius) and pressure (hundreds of bars), as well as shear stresses. These reactive intermediates are essential for producing hydrogen gas from water and facilitating other chemical changes [3]. Regulated generation of these species under ultrasonication provides a clean, sustainable method of producing hydrogen, eliminating the need for traditional, energy-intensive procedures [4]. Ultrasonication processes can produce hydrogen and hydrogen peroxide, two essential compounds with significant applications [5,6]. For instance, hydrogen peroxide is a versatile, eco-friendly oxidant used in many industries. Additionally, hydrogen is a clean fuel with great potential for transitioning to renewable energy sources.

Ultrasonication methods show promise as a way to produce hydrogen. However, they are often limited by low yields of a few tens of µM/min due to inefficient energy transfer and the significant energy required for effective cavitation [7,8]. Due to these limitations, more effective alternative methods must be investigated to enhance hydrogen generation [9]. One feasible option is ultrasonication-hybrid processes, which integrate sonochemistry with several catalytic methods. These systems leverage the synergistic effects of ultrasonication and various catalytic processes, including US/catalysis, US/photocatalysis, and US/electrocatalysis. Each of these processes offers unique benefits [[10], [11], [12]]. Among these, US/electrocatalysis, which combines electrolysis and ultrasonication to improve hydrogen generation, has seen the greatest breakthroughs [4,7,13,14]. Ultrasonication enhances mass transfer, decreases bubble formation on electrode surfaces, and accelerates electron transfer in electrolysis by combining physical and chemical processes [15]. These impacts result in higher overall effectiveness, reduced overpotentials, and faster reaction rates. Due to its advantages over classical electrolysis techniques, US/electrocatalysis is an encouraging strategy for large-scale hydrogen generation [15]. Thus, combining ultrasonication with electrolysis effectively overcomes the limitations of conventional sonolytic methods and significantly increases hydrogen generation yields [15].

This study aims to deliver an update on the latest progresses in ultrasonication-hybrid techniques for hydrogen generation, with a focus on the US/electrochemical process. It will demonstrate how integrating ultrasonication with different catalytic processes can augment the efficacy of hydrogen generation. Additionally, the study will thoroughly cover the US/electrochemistry process. This field applies ultrasonication to electrochemical techniques to optimize physical and chemical aspects and improve performance. Specifically, the paper will explain how ultrasonication enhances mass transfer, reduces overpotentials, and accelerates electrochemical reactions. These effects increase hydrogen yield and improve energy effectiveness.

## 2 Hydrogen generation via US/electrochemical technique

### 2.1 Electrolysis

Electrochemistry is the study of the relationship between electrical and chemical phenomena, with a particular focus on electron transport at the interface between an electrode and a solution [16]. The structure of this interface significantly impacts current responsiveness [17,18]. According to models, this interface consists of an electric double layer of solvated ions that counterbalance the electrode's charge due to coulombic attraction. Consequently, a strong electric field is concentrated across an area only a few nanometers thick [17]. More advanced models depict the interface as two charge-separated layers: the outer Helmholtz plane, which contains solvated cations that extend into the diffusion layer; and the inner Helmholtz plane, consisting of sorbed solvent molecules and anions. Thermal motion and coulombic ordering struggle inside this layer [17]. The effectiveness of electrode reactions depends on diffusion rates, reactant and product concentrations, electron-transfer kinetics, and electrode characteristics.

Water electrolysis is an essential method for producing hydrogen. The technique encompasses two half-cell reactions: the hydrogen evolution reaction (HER) at the cathode (reactions (1), (2) and the oxygen evolution reaction (OER) at the anode (reactions (3), (4) [19].

(1) $$\text{Acidic media}:\;2{\text{H}}^{+}+\;\;2\bar{\text{e}}\to \;{\text{H}}_{2}\;\left({\text{E}}^{\text{o}}\;=\;0.0\;\text{V}/\text{NHE}\right)\;$$

(2) $$\text{Alkaline media}:\;2{\text{H}}_{2}\text{O}\;+\;2\bar{\text{e}}\to \;{\text{H}}_{2}\;+\;2{\text{OH}}^{-}\left({\text{E}}^{\text{o}}=\;-\;0.828\;\text{V}/\text{NHE}\right)\;$$

(3) $$\text{Acidic media}:\;{\text{H}}_{2}\text{O}\;\to \;2{\text{H}}^{+}\;\text{+ }1/2\;{\text{O}}_{2}\;+\;2\bar{\text{e}}\;\left({\text{E}}^{\text{o}}=+\;1.229\;\text{V}/\text{NHE}\right)\;$$

(4) $$\text{Alkaline media}:\;2{\text{OH}}^{-}\;\to \;1/2\;{\text{O}}_{2}\;+\;{\text{H}}_{2}\text{O}\;+\;2\bar{e}\;\left({\text{E}}^{\text{o}}\;=\;+\;0.401\;\text{V}/\text{NHE}\right)\;$$

The four principal categories of water electrolysis processes are solid oxide electrolysis cell (SOEC), anion exchange membrane water electrolyzer (AEMWE), proton exchange membrane water electrolyzer (PEMWE), and alkaline water electrolyzer (AWE). SOEC can function at temperatures of overhead 700 °C. Yttrium-stabilized zirconia (YSZ) serves as the solid electrolyte, and the ionic species are oxide ions (O^2–^) [20]. Cost-effective non-precious metal catalysts like Ni, Co, or Fe can be used as the main catalytic component in AEMWE by using anion exchange membranes made from divinylbenzene (DVB) that preferentially conduct OH^–^. AEMWEs function at temperatures lower than 80 °C [21]. In contrast to its alkaline equivalent, the PEMWE uses a solid perfluorosulfonic acid (PFSA) membrane as the electrolyte. Hydrogen cations (H^+^) are the ionic species. Usually, PEMWEs operate at temperatures below 80 °C [20]. The electrolyte of an AWE is a liquid solution of either sodium hydroxide or potassium hydroxide. The ionic species in this cell are hydroxyl ions, and it operates at temperatures lower than 80 °C [20].

• ***Overpotentials***

There are three crucial kinetic phases involved in electrode operations in an electrolysis cell. First, ions migrate from the bulk liquid to the electrode surface via mass transport. Second, ionic discharge occurs. At the electrode surface, electrons move to or from the ions. Third, the released atoms then undergo conversion, changing into a more stable state.

These actions lead to many kinds of overpotentials. Slow mass transport (Stage 1) results in restricted ion availability close to the electrode, which causes concentration overpotential (η_C_). Activation overpotential (η_A_) linked to energy barriers for stable product synthesis and ionic discharge (Stages 2 and 3), especially for metal deposition and gas evolution. Ohmic overpotential (η_R_) caused by the electrolyte's resistance and ion depletion during discharge.

The following equation gives the overall overpotential, η:

(5) $$\eta_{=}\eta_{c(cathode)}+\eta_{a(anode)}=\eta_{C,c}+\eta_{A,c}+\eta_{R,c}+\eta_{C,a}+\eta_{A,a}+\eta_{R,a}$$

Substantial overpotentials, particularly for the oxygen evolution reaction (OER), increase the voltage required for water splitting beyond what is needed for thermodynamic breakdown. Furthermore, energy consumption increases due to the ohmic voltage drop across the cell caused by gas bubbles in the solution and on the electrode surface [22]. In water electrolysis, the combined resistance can be written as follows [23]:

(6) $$\Sigma R=R_{e}+R_{m}+R_{b}+R_{c}$$

where R_b_ is the bubble resistance, R_m_ denotes the membrane resistance, R_e_ represents the electrolyte resistance, and R_c_ represents the circuit resistance. Improvements in wire connections and membrane fabrication techniques can reduce R_m_ and R_c_, which remain essentially constant. Meanwhile, R_b_ and R_e_ are variable.

Dispersing gas bubbles (H_2_ and O_2_) in the electrolyte decreases conductivity and creates bubble resistance, also known as ‘bubble overpotential’. Resistance increases further when bubbles form on the electrode surface since the bubbles obstruct the electric field [23,24]. The following expression represents the total cell voltage, V_cell_, for water electrolysis:

(7) $$Vcell\;=\;\left|E_{c}-E_{a}\right|+\;I\times \;\sum R\;=\;E_{\mathrm{rev}}\;+\;\left|\eta_{a}\right|\;+\;\left|\eta_{c}\right|\;+\;I\;\times \;\left(R_{e}\;+\;R_{m}\;+\;R_{b}\;+\;R_{c}\right)$$

where I is the applied current, E_rev_ is the reversible Nernst potential, η_a_ and η_c_ are the anode and cathode overpotentials, respectively, and E_c_ (or EHER) is the cathode potential for HER and E_a_ (or EOER) is the anode potential for OER.

The total cell voltage's substantial reliance on the ohmic voltage drop and overpotential is evident in Eq. (7). Water electrolysis has a predicted breakdown voltage of +1.229 V/RHE. However, water electrolysis cells consume more energy than the theoretical minimum in practice because they operate at voltages varying from +1.70 to +2.5 V [23]. Therefore, reducing η_a_, η_c_, and ΣR is essential to increasing efficiency.

• ***Energy Usage and Ultrasonication***

Table 1 compares the efficiency of hydrogen generation and energy consumption for different aqueous electrolytes. Studies have shown that using ultrasonication can diminish energy utilization and increase the efficiency of hydrogen generation, making ultrasonication a viable alternative to industrial electrolyzers. For instance, the efficiencies of potassium hydroxide and sodium hydroxide electrolytes increased by 3.26 and 5.31 %, respectively, when subjected to 20 kHz ultrasonication. Interestingly, despite its stronger conductivity and potential for hydrogen generation, the efficiency gain for potassium hydroxide was less noticeable. This disparity was attributed to the cell design, in which the capture of produced gases was constrained by the tube's diameter. In the case of potassium hydroxide, large amounts of gas escaped capture by dispersing into the surrounding medium.

**Table 1:** Energy usage and hydrogen generation effectiveness through water electrolysis in diverse aqueous solutions.

System	Theoretical Energy usage (kWh/m^3^ H_2_)	Practical energy usage (kWh/m^3^ H_2_)	Hydrogen generation efficiency (%)
Commercial and industrial electrolyzers [38], [58], [59]	2.94	∼4.49–5.39	<73
Seawater [23], [60]	2.94	5.03	58.57
Brine electrolysis [23], [60]	2.94	5.53	53.25
0.1 M NaOH [23], [38]	2.94	6.15	78.44
0.1 M NaOH with 20 kHz ultrasonication [23], [38]	2.94	5.53	83.75
0.1 M KOH [23], [38]	2.94	6.03	77.1
0.1 M KOH with 20 kHz ultrasonication [23], [38]	2.94	5.12	80.36
0.1 M NaOH [23], [43]	2.94	∼8.5	60–75
0.1 M NaOH with 60 kHz ultrasonication [23], [43]	2.94	∼7.5	80–85

### 2.2 US/electrochemistry

To enhance the rate and efficiency of reactions, sonoelectrochemistry (US/electrochemistry) integrates electrochemistry with ultrasonication [17]. This approach was first applied to water electrolysis using a platinum electrode, where ultrasonication resulted in lower cell voltages and faster reaction rates compared to conventional electrolysis. The fundamental processes of US/electrochemistry involve harsh environments caused by cavitation and acoustic streaming. These occurrences can introduce novel electrochemical processes or improved reaction routes [25]. Studies have shown that ultrasonication affects homogeneous systems in the bulk electrolyte solution and heterogeneous systems at the electrode–electrolyte interface [25,26].

**Fig. 1:**
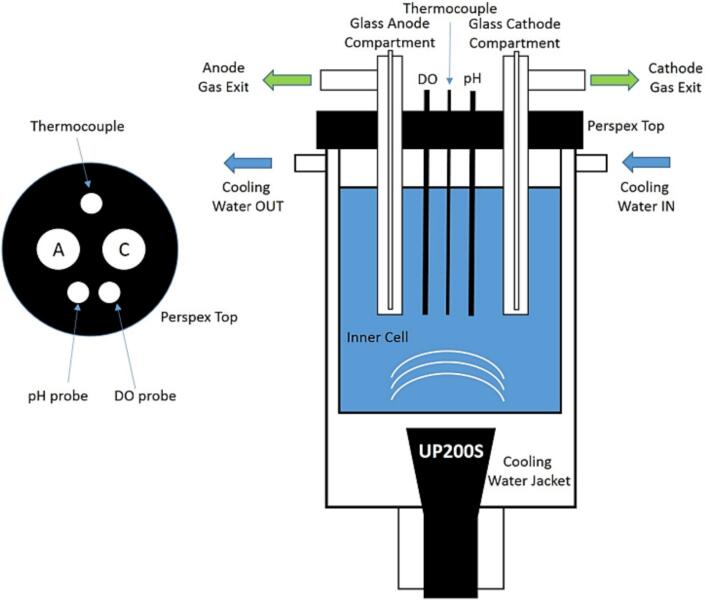
Schematic illustration of a typical experimental configuration for a US/electrochemical reactor [23]. In this setup, which employs a 'face-on' geometry, the ultrasonication probe (UP200S) is oriented toward the anode and cathode to promote the generation of oxygen and hydrogen in alkaline solutions. To avoid contamination, the ultrasonication probe does not come into direct contact with the electroanalytic.

Fig. 1 elucidates a schematic illustration of a typical US/electrochemical configuration. Ultrasonication can be applied using a probe or an ultrasonication bath. Depending on the configuration, the probe can be directly immerged in the electrolyte or kept outside the electrochemical cell. For both configurations, position the sonolytic horn so that it is facing the surface of the working electrode. This alignment is referred to as ‘face-on’ configuration. This alignment optimizes cavitation and acoustic phenomena, thereby enhancing the reaction and ensuring effective energy transfer to the electrode surface.

### 2.3 Electrochemical processes enhanced by ultrasonication

US/electrochemistry combines electrochemistry and acoustic cavitation, and is used in many fields [27]. Ultrasonication improves these processes through physical and chemical effects, providing numerous advantages.

**Fig. 2:**
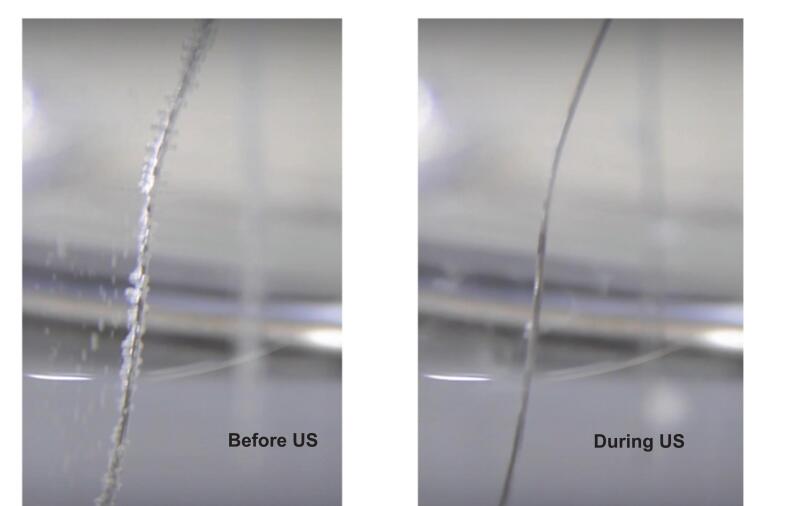
Hydrogen gas flux on Pt wire before and during ultrasonication (US) [23].

**Fig. 3:**
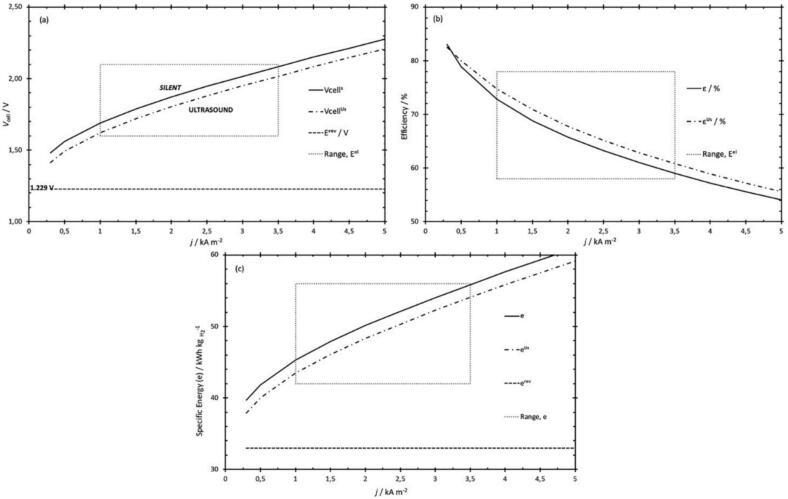
Ultrasonication effect on (a) cell voltage (b) efficiency (ε) and (c) specific energy for hydrogen production (UsA: Ultrasonication-assisted) [23].

The mechanical effects of ultrasonication, including microjets, acoustic streaming, and micro-mixing, are crucial for enhancing electrochemical processes. These effects speed up mass transfer by reducing the diffusion layer thickness and creating a cleaning effect. Photographic observations of hydrogen bubbles on a platinum wire electrode (Fig. 2) demonstrate ultrasonication's cleaning impact, improved ion transport between double layers, local activation of the electrode surface, and augmented current efficiency during electrochemical processes [27]. Fig. 3 shows that Islam et al. [23] found that a combination of variables, such as augmented ion concentration and bubble disengagement at the electrode surface, can increase electrolytic efficiency by 15–20 %.

**Fig. 4:**
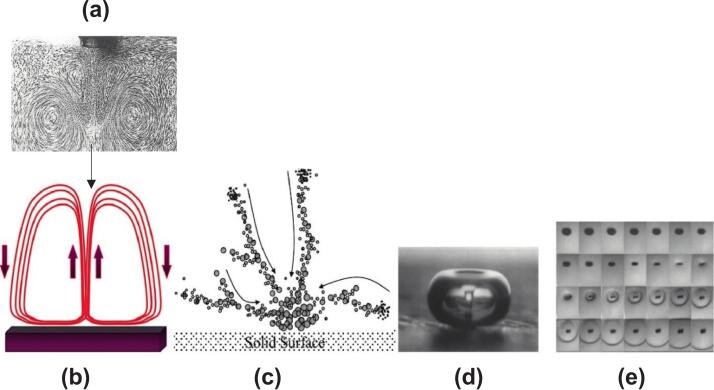
(a) Acoustic streaming (oscillator is located at the bottom and liquid surface is at the top), (b) schematic of acoustic streaming, (c) microstreamers, (d) microjet and (e) shockwaves generated by ultrasonication and acoustic cavitation [63].

Acoustic streaming is a constant fluid flow caused by the transmission of ultrasound. This phenomenon occurs due to nonlinear, time-averaged effects of changes in the pressure field. These effects generate fluid movement in three areas: the reactor walls, the bulk solution, and the boundary layers. Fig. 4a shows streak photos of Eckart streaming (schematized in Fig. 4b) a few centimeters from an sonolytic transducer [28]. Microstreaming results from the vorticity generated by an oscillating body in a fluid's boundary layer. Examples include cavitation bubbles and solid microparticles (Fig. 4c). Streaming strength is inversely affected by viscosity and ultrasound speed [29]. Ultrasonication intensity, emitter surface area, and medium attenuation coefficient also impact streaming strength. Acoustic streaming increases the mass transfer of electroactive species to the electrode surface by reducing the diffusion boundary layer [25,30].

Another mechanical result is the formation of microjets (Fig. 4d). This occurs when cavitation bubbles collapse in close proximity to solid surfaces. These jets propel fluid toward the electrode surface at speeds attaining 200 m/s. This improves mass transfer, cleans the electrodes, and prevents fouling [31]. A related phenomenon, microstreaming, enhances the motion of confined fluids around solid particles or pulsing bubbles, thereby facilitating mass transfer. Bubble collapse produces shock waves (Fig. 4e) that degrade electrode surfaces, thereby increasing current efficiency and reaction rates [32]. As these high-pressure shock waves pass through the medium, they clean and activate the electrodes.

Ultrasonication-induced cavitation, produced by ultrasonication of the electrolyte, generates chemically active radicals (e.g., ^•^OH, HO_2_^•^, and O^•^). These phenomena are acknowledged as the chemical effects [25]. These radicals can drive novel reaction pathways. However, the chemical effects of US/electrochemistry are often limited by lower bubble temperatures near electrode surfaces because bubble symmetry breaks down during collapse. The hotspot theory attributes the generation of extreme pressures and temperatures to bubble collapse; however, these conditions are less significant in the vicinity of electrodes (due to bubble asymmetry), thereby diminishing the extent of chemical effects [33].

Integrating ultrasonication into electrochemistry has several advantages. For instance, it can disrupt the Nernst diffusion layer, remove gas bubbles from electrode surfaces, enhance kinetics, degas the solution, and facilitate mass transport through the double layer. Ultrasonication can also clean and activate electrode surfaces, decrease electrode overpotential, and improve electrodeposit quality. However, obstacles to the widespread use of US/electrochemistry include optimizing electrode placement and cell design, as well as managing the length between the electrode and the ultrasonication horn. Furthermore, achieving optimal system efficiency necessitates precise management of ultrasonication parameters, such as frequency and acoustic power.

### 2.4 Hydrogen generation via US/electrochemistry technique

Table 2 summarizes the key findings and operating parameters of notable studies on US/electrochemical hydrogen generation. The most intriguing findings are discussed in the following section.

**Table 2:** Main works focusing on the US/electrochemical generation of hydrogen.

Work	Operating conditions	Main observations
Pollet et al [44]	Freq. 26 kHz. I_acoustic_ = 75 W/cm^2^. T_liq_ = 25 °C. Working Electrode: platinum (Pt) polycrystalline disc/wire. Counter electrode: Pt wire sealed in a glass tube. Reference electrode: a homemade reversible hydrogen electrode (RHE). [H_2_SO_4_] = 0.5 M. Electrochemical technique: cyclic and linear sweep voltammetry.	− The current density was enhanced by 250 % (from ∼ 70 to ∼ 170 mA/cm^2^) by increasing the acoustic power to 26.20 W (75 W/cm^2^). − Ultrasonication had minimal impact on the “actual” Pt surface area and roughness factor. − Ultrasonication did not significantly alter the mechanism of HER; rather, it increased currents at the Pt surface by effectively removing hydrogen bubbles.
Cataldo [34]	Freq. 30 kHz. I_acoustic_ = 1–2 W/cm^2^. Electrodes: carbon electrode rod. [NaCl]_0_ = 6 M. [HCl] = 6 M. [NaCl/HCl] = 5/1.1 M.	− Ultrasonication create a powerful degassing effect (benefiting both hydrogen and chlorine production) through bubbles coalescence and their mechanical stripping.
McMurray et al. [35]	Freq. 20 kHz. I_acoustic_ = 26 W/cm^2^. T_liq_ = 30 °C. Electrodes: Graphite. [Na_2_SO_4_] = 0.7 M. Gas: Ar. Electrochemical technique: linear sweep voltammetry.	− Applying ultrasonication significantly increased the generation of both oxygen and hydrogen gases. −The use of ultrasonication enhanced mass transport at the cathode, consequently boosting the oxygen reduction reaction − A significant improvement in hydrogen evolution compared to oxygen evolution, which was attributed to a reduction in the activation overpotential (η_a_).
Budischak et al [42]	Freq. 42 kHz. P_electric_ = 300 W. Working and auxiliary electrodes’ material: platinum. Reference electrode: standard calomel electrode (SCE). [KOH]_0_ = 2 M. Saturating gas: Ar. Electrochemical technique: linear scan voltammetry and chronoamperometry.	− The obtained findings highlight the significant efficiency improvements that ultrasonication can bring to an electrolyzer, particularly at intermediate current densities.
Lin et al [41]	Freq. 133 kHz. P_acoustic_ = 225–900 W. [KOH] = 10–40 wt%. Electrode distance: 2, 5, and 10 mm. Electrodes’ material: Nickel. Electrochemical technique: Electrochemical impedance spectroscopy (EIS)	− A current density difference of 240 mA/cm^2^ between water electrolysis conducted with and without ultrasonication (225 W) was retrieved under conditions of room temperature, a 2 mm electrode gap, 4 V cell voltage, and 40 wt% KOH. − The US/electrolytic process needed 3.5 kW less power and showed a 15 % increase in economic power efficiency. − Ultrasonication improved the removal of bubbles from the electrodes (cleans the surface of large bubbles that can clog active sites), increased mass transfer, and reduced concentration polarization.
Li et al [43]	Freq. 60 kHz. P_electric_ = 50 W. [NaOH] = 0.1, 0.5, 1 M. T_liq_ = 30 °C. Working and counter electrodes: RuO_2_ and IrO_2_ plated Ti. Reference electrode: Ag/AgCl. Electrochemical approach: linear sweep voltammetry (LSV) and galvanostatic polarization.	− The values of the cell voltage reduction for 0.1, 0.5 and 1.0 M NaOH were about 320, 100 and 75 mV at 200 mA/cm^2^, respectively. − The amount of H_2_ gas bubbles produced by ultrasonication increased by approximately 5–18 % with the application of high current densities. − The hydrogen generation results indicated that, under the experimental conditions, the energy savings achieved with the ultrasonic field were reduced by approximately 10–25 %.
Zadeh [38]	Freq. 20 kHz. P_electric_ = 750 W. [NaOH] = 0.1 M. [KOH] = 0.1 M. Volume = 1 L. T_liq_ = 25 °C. Electrodes’ material: Nickel.	− The average production efficiency of the electrolysis cell was 78 %. − Ultrasonication led to a 4.5 % increase in production efficiency and a 1.3 % improvement in energy efficiency. − By removing bubbles from the electrode and electrolyte, ultrasonication facilitates contact (trough the preparation of electrode’s surface) and enhances the efficiency of hydrogen production.
Symes [39]	Freq. 20 kHz. I_acoustic_ = 20.7 W/cm^2^. Volume: 200 mL. [NaOH]_0_, [NaCl]_0_, [H_2_SO_4_]_0_ = 0.1–1 M. T_liq_ = 25 °C. Electrodes’ material: carbon.	− Ultrasonication was noted to decrease both the anodic and cathodic overpotentials by promoting gas removal at the electrode surface through cavitation and enhancing mass transfer. − Despite this effect, ultrasonication did not elevate the rate of hydrogen production.
Walton et al. [36]	Freq. 38 kHz. P_electric_ = 150 W. [H_2_SO_4_] = 1 M. T_liq_= NI. Electrodes’ (working and counter) material: Platinum. Reference electrode: hydrogen reference electrode. Electrochemical approach: cyclic voltammetry.	− Ultrasonication significantly boosts the rates of both hydrogen and chlorine evolution on platinized platinum. − The researchers observed a 2.1-fold increase in current compared to without ultrasonication. − The main reason for the increased current was the removal of hydrogen bubbles from the electrode surface by ultrasonication, not enhanced diffusion of H^+^ ions.
Li et al. [40]	Freq. 25.3 and 33.3 kHz. P_electric_ = NI. [NaOH] = 0.4 M. T_liq_ = NI. Electrodes’ material: pure-graphite. Reference electrode: saturated calomel electrode (SCE). Electrochemical approach: chronopotentiometry method.	− Notably, a 25.3 kHz ultrasonication resulted in a larger decrease in cell voltage (reduced by 150 to 300 mV), when the current was between 20 and 40 mA. − The observed decrease in cell voltage was linked to the elimination of gas bubbles from the surface of the electrode.
Kerboua and Merabet [45]	Freq. 40 kHz. P_ac_ = 8.13 W. Volume: 300 mL. [KOH] = 25 % w/w. T_liq_ ≈ 28 °C. Electrodes’ material: Nickel.	− The integration of ultrasonication into the electrolysis process yielded an average enhancement of 3.93 % in hydrogen production rate. − Experimental and computational assessments of hydrogen production rates revealed a negligible contribution from sonochemistry. Instead, the study attributed the effects of ultrasonication to the actions of shockwaves and microjets. − A reduction in electrode bubble coverage from 76 % to 42 % was observed, leading to a 7.2 % decrease in Ohmic resistance and a substantial 62.35 % decrease in bubble resistance.
Merabet and Kerboua [46]	Freq. 40 kHz. P_electric_ = 60 W. Volume: 300–350 mL. [KOH, NaOH] = 2.67, 3.56, 4.46, 5.35, 6.24, 7.13 M. T_liq_ = 27, 40, 45, 50, 55 and 60 °C. Electrodes’ material: stainless steel 304, nickel, nickel foam and graphite.	− Ultrasonication enhanced both the efficiency and kinetics of hydrogen production, with particular improvements observed in continuous operation. − Employing nickel foam electrodes in a KOH electrolyte under optimal conditions, the continuous application of ultrasonication at ambient temperature led to a 7.78 % increase in cell current, accompanied by a 64 % reduction in bubble resistance.
Merabet and Kerboua [61]	Freq. 40 kHz. P_electric_ = 60 W. Volume: 300 mL. [KOH] = 4.46 M. T_liq_ = 25 °C. Electrodes’ material: Nickel.	The integrated approach (PV system and sono-electrolyzer) revealed a 76 % decrease in bubble resistance when employing indirect continuous ultrasonication compared to silent conditions, and a 52 % reduction relative to pulsed sonication.
Foroughi et al [7]	Freq. 408 kHz. P_ac_ ≈ 54 W. Volume: NI. [KOH] = 30 % w/w. T_liq_ = 24, 40, and 60 °C. Electrodes’ type: Raney-Ni. Electrochemical approach: Linear sweep voltammetry (LSV) and electrochemical impedance spectroscopy (EIS).	− Under silent conditions: The electrocatalytic performance of Raney-Ni for both HER and OER depends on the electrolyte temperature, where elevated temperatures facilitated higher current densities at reduced overpotentials. − Under ultrasonication: the HER activity of Raney-Ni was enhanced at lower temperatures (e.g., 25 °C), the influence of ultrasonication on the OER was negligible. − Ultrasonication improves HER by efficiently removing gas bubbles from the electrode.

Cataldo's groundbreaking work [34] is a seminal investigation into the effects of ultrasonication on electrolysis techniques. While conducting electrolysis with sodium chloride and hydrochloric acid, he examined the effects of 30 kHz ultrasonication with an acoustic intensity of 1–2 W/cm^2^. The study evaluated the generation of chlorine gas and hydrogen at the anode and cathode, respectively, under ultrasonicated and silent conditions. The results revealed substantial enhancements due to ultrasonication, particularly in the anode's generation of chlorine gas. These improvements were attributed to increased degassing, which promoted bubble detachment and coalescence, as well as improved mass transfer via acoustic cavitation. This accelerated electrochemical techniques. Ultrasonication improved electrolysis efficiency 2.81-fold at higher current densities in a 5 M sodium chloride and 1.1 M hydrochloric acid solution compared to the absence of ultrasonication. However, the effects of lower electrolyte concentrations were not examined because the study focused on high concentrations. Examining electrolyte characteristics more thoroughly may provide additional information on improving US/electrochemical performance.

McMurray et al. [35] investigated the impact of 20 kHz ultrasonication (26 W/cm^2^) on a US/electrolytic process employing a titanium sonotrode, graphite electrodes, and a 0.7 M sodium sulfate solution at pH 7. Ultrasonication greatly increased the generation of hydrogen and oxygen gas, with hydrogen generation showing the greatest enhancement. Improved mass transfer brought about by ultrasonication decreased the activation overpotential (η_a_) for hydrogen on graphite electrodes. However, the study also found that enhanced cavitation accelerated rates of hydrogen evolution and oxygen reduction. This could hasten electrode degradation despite demonstrating how ultrasonication might increase electrolysis efficiency. Further investigation into ultrasonicator placement and operating settings is required to overcome these obstacles.

In contrast to quiet settings, Walton et al. [36] demonstrated that applying 38 kHz ultrasonication in a sonolytic bath greatly increased the current during hydrogen generation from a 1 M sulfuric acid solution at a platinum electrode. The increase was 2.1-fold. The researchers attributed this increase to sonolytically removing hydrogen bubbles from the electrode surface rather than to increased H^+^ diffusion, despite the reversible nature of the hydrogen ion reaction at the electrode. Based on this work, researchers [37,38,39] used various electrodes (e.g., platinum, 316 stainless-steel, industrial carbon, and glassy carbon) to study ultrasonication-assisted hydrogen generation in mildly acidic (sulfuric acid) and alkaline (sodium hydroxide and potassium hydroxide) solutions. Their research indicates that ultrasonication can lower the total reaction overpotential by +100 to +400 mV dependent on the acoustic power, electrode material, electrolyte content, and solution type. For example, 20 kHz ultrasonication decreased the overpotential from +1.30 V to +0.92 V and the decomposition cell voltage from +2.52 V to +2.14 V in a 0.1 M potassium hydroxide solution [37,38]. By investigating the impact of various factors, including acoustic power, electrolyte content, and electrode materials, Prof. Pollet's research group has significantly advanced ultrasonication-assisted hydrogen generation.

Li et al. [40] investigated the impact of ultrasonication on the formation of hydrogen in a 0.4 M sodium hydroxide solution. They produced the ultrasound waves using a random waveform generator, a power amplifier, and piezoelectric crystals. According to their results, the cell voltage diminished by 150–300 mV at 25.3 kHz across a current interval of 20–40 mA. This voltage decrease occurred thanks to the gas bubbles elimination from the electrode surface. Interestingly, the voltage diminution did not change when the ultrasonication frequency increased to 33.3 kHz. Photographic analysis of gas bubble formation revealed that the voltage decrease was caused by a combination of mechanical and thermal effects, as well as the resonant effect.

Lin and Hourng [41] examined the impact of 133 kHz and 225–900 W ultrasonication on hydrogen formation via water electrolysis. In contrast to other research in this area, they used high-frequency ultrasonication, shifting the focus toward the chemical impacts of ultrasonication. Moreover, their study was the first to apply electrochemical impedance spectroscopy (EIS) to investigate polarization impedance patterns in sonolytic water electrolysis. At a cell voltage of 2 V, they found that ultrasonication increased activity and concentration impedances, accelerating the disengagement of hydrogen bubbles. Using a 2-mm electrode gap, a 4-V cell voltage, and a 40 wt% potassium hydroxide solution at room temperature, they observed a 240 mA/cm^2^ increase in current density in ultrasonication-assisted water electrolysis (225 W) compared to conventional water electrolysis. The paper concluded that ultrasonication reduced power consumption by 3.5 kW and improved economic power efficacy by 15 %. These enhancements were attributed to decreased concentration polarization, enhanced mass transfer, and bubble elimination caused by ultrasonication. Budischak et al. [42] studied the impacts of 42 kHz and 300 W ultrasonication on hydrogen formation in a 2 M potassium hydroxide solution with platinum electrodes. They observed, particularly at intermediate current densities, significant improvements in electrolyzer efficiency through reduced overpotential and enhanced mass transport via bubble detachment from the electrode surface.

**Fig. 5:**
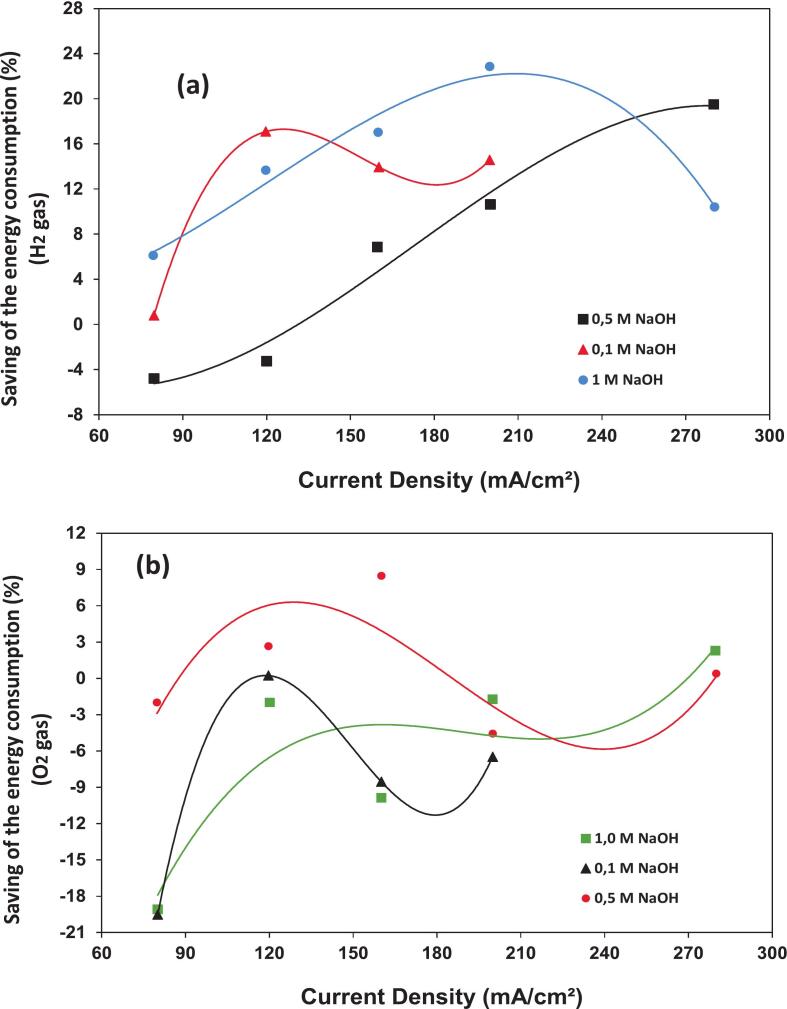
The energy saving of (a) H_2_ and (b) O_2_ generation in the presence of ultrasonication [f = 60 kHz, P_E_ = 50 W] in 0.1, 0.5, and 1.0 M NaOH for a constant current density [43].

Li et al. [43] examined the influence of 60 kHz and 50 W ultrasonication on water electrolysis using 0.1, 0.5, and 1 M sodium hydroxide solutions. Their research showed that electrolysis of water assisted by ultrasonication significantly increased energy efficiency. Measurements of cell voltage, energy usage, and efficiency throughout the electrolysis system revealed this improvement. Maintaining an identical current density revealed that the voltage difference between ultrasonicated and silent conditions decreased as electrolyte content increased. Specifically, ultrasonication substantially reduced the cell voltage, with decreases of approximately 320 mV, 100 mV, and 75 mV at 200 mA/cm^2^ for 0.1, 0.5, and 1 M NaOH, respectively. Ultrasonication increased hydrogen formation effectiveness by 5–18 % at high current densities. However, variations in gas bubble behavior caused a slight decrease in oxygen formation efficiency. For hydrogen formation with a specific electrolyte content and high current density, ultrasonication yielded energy savings of 10–25 % (Fig. 5a). Conversely, the energy required to produce oxygen remained essentially unchanged, regardless of whether ultrasonication was used (Fig. 5b). Islam et al. [13] attributed ultrasonication's impact on electrolytic hydrogen generation to improved mass transfer and electrode cleaning induced by microjets and microstreaming. This conclusion is reliable with results reported in the literature. However, at low electrolyte contents and high current densities, ultrasonication decreased cell voltage significantly. Additionally, Islam et al. [13] proposed that degassing and/or changes to the electrode surface caused the total potential decrease.

**Fig. 6:**
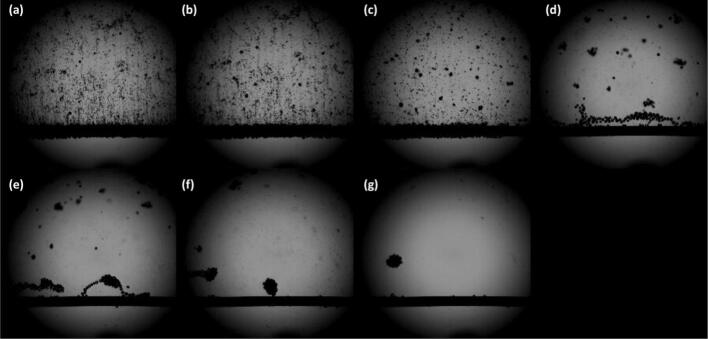
Hydrogen generation on a Pt wire in the absence (a) and presence of ultrasonication (26 kHz, 75 W/cm^2^, (b)-(g)). The applied potential was set at − 1.30 V/RHE. (a) 0 μs, (b) 100 μs, (c) 200 μs, (d) 300 μs, (e) 400 μs, (f) 500 μs, (g) 600 μs. The time between each image is 10^-4^ s (100 μs) filmed at 10,000 frames per second [44].

Earlier research indicates a significant gap in kinetic analysis that could clarify how ultrasonication affects the HER process and Tafel parameters in slightly acidic and alkaline electrolytes. Recently, Pollet et al. [44] examined the impacts of 26 kHz ultrasonication, up to 75 W/cm^2^, on HER in a 0.5 M sulfuric acid solution using a platinum polycrystalline disc electrode. Using ultra-fast camera imaging and linear and cyclic sweep voltammetry, the researchers investigated the generation of hydrogen bubbles on a platinum wire under ultrasonicated and silent conditions. They discovered that, as ultrasonication amplitude increased, the current density at an electrode potential of –0.10 V/RHE rose from approximately 70 mA/cm^2^ at 0 % to about 170 mA/cm^2^ at 100 %. This resulted in a 2.5-fold intensification in current density at the maximal ultrasonication power of 29.20 W. The roughness factor and the ‘actual Pt surface area’, which specify the microscopic surface area accessible for electron transport, were not significantly impacted by ultrasonication under these conditions. According to the results, ultrasonication enhanced currents by eliminating hydrogen bubbles from the platinum surface rather than substantially altering the HER mechanism (Fig. 6).

The influence of sonolysis on alkaline electrolysis in a membraneless H-cell that was subjected to 40 kHz and 60 W of indirect and continuous ultrasonication was examined by Kerboua and Merabet [45]. Using experimental data, the researchers developed a mathematical model that incorporated electrochemical resistances and the dynamic behavior of cavitation bubbles. They then used the model to simulate the performance of a US/electrolyzer. The experimental outcomes demonstrated that integrating sonication led to an average increase of only 3.93 % in the H_2_ production rate, accompanied by a corresponding 2.76 % improvement in energy conversion efficiency. The 40 kHz ultrasonication frequency favors the physical impacts of cavitation (i.e., shock waves and microjets); thus, these results should be confirmed at higher frequencies. Ohmic resistance decreased by 7.2 %, and bubble resistance decreased by 62.35 % as electrode bubble coverage decreased from 76 % to 42 %. In follow-up research [46], the research team investigated the impact of ultrasonication on a water electrolysis system while considering factors such as electrode materials, temperature, electrolyte concentration, and salt type. Continuous ultrasonication at room temperature with nickel foam electrodes in potassium hydroxide diminished bubble resistance by 64 % and augmented cell current by 7.78 %. In terms of kinetic and energy effectiveness, continuous ultrasonication outperformed pulsed ultrasonication. They used a membraneless US/electrolyzer driven by photovoltaics to generate hydrogen with both techniques. Despite changes in solar radiation, the PV panel maintained a constant cell voltage of 5.957 V (±0.1 %). Compared to quiet settings, continuous ultrasonication reduced bubble resistance by 76 %, while pulsed ultrasonication reduced it by 52 %.

Foroughi et al. [7] investigated the HER and OER at a Raney-Ni mesh electrode in a 30 wt% potassium hydroxide solution using 408 kHz ultrasonication. The investigation was concucted at electrolyte temperatures of 25, 40, and 60 °C without and with ultrasonication (100 % acoustic amplitude, approximately 54 W) (Table 3, Table 4). Raney-Ni electrocatalytic performance demonstrated temperature dependency in the absence of ultrasonication: higher temperatures resulted in greater current densities at lower overpotentials. Ultrasonication settings did not significantly affect the OER but increased the HER activity at lower temperatures (e.g., 25 °C). Remarkably, as the electrolyte temperature increased, the amplification of both processes decreased. This is presumably engendered by the unfavorable impact of higher temperatures on acoustic cavitation. The efficient elimination and dispersion of gas bubbles from the electrode surface into the electrolyte primarily accounted for the improved HER performance, even at a high frequency of 408 kHz.

**Table 3:** Comparison of Tafel slopes (b), exchange current densities (j_0_), overpotential (ƞ) at –300 mA/cm^2^ and the difference between the overpotentials in the absence and presence of ultrasonication (ΔE) for the HER on Raney-Ni in 30 wt-% aqueous KOH solution at 25, 40 and 60 ^◦^C [7].

Temperature (^◦^C)	Ultrasonication amplitude	b* (mV/dec)	j_o_ (mA/cm^2^)	Overpotential at –300 mA/cm^2^ (mV)	Δη (mV)
25	0 (silent) 100 %	113 105	0.52 0.77	−308 −274	34
40	0 (silent) 100 %	128 135	8.38 13.07	−203 −190	13
60	0 (silent) 100 %	130 144	52.78 83.73	−140 −135	5

*-180 ≤ η ≤ -300 mV.

**Table 4:** Comparison of Tafel slopes (b), exchange current densities (j_0_) and overpotential (ƞ) at +300 mA/cm^2^ for the OER on Raney-Ni in 30 wt-% aqueous KOH solution at 25, 40 and 60 ^◦^C [7].

Temperature °(C)	Ultrasonication amplitude	b (mV/dec) at low overpotential*	b (mV/dec) at high overpotential^**^	j_o_ (mA/cm^2^) at low overpotential	j_o_ (mA/cm^2^) at high overpotential	Overpotential at +300 mA/cm^2^ (mV)
25	0 (silent) 100 %	39 42	90 74	1.35 × 10^-5^ 5.51 × 10^-5^	0.12 0.038	302 290
40	0 (silent) 100 %	39 41	84 101	4.00 × 10^-5^ 7.9 × 10^-5^	0.18 0.54	274 278
60	0 (silent) 100 %	39 42	80 102	12.00 × 10^-5^ 45.00 × 10^-5^	0.30 1.25	247 248

*200 ≤ η ≤ 250 mV.

**250 ≤ η ≤ 300 mV.

## 3 Ultrasonication-Driven techniques

**Fig. 7:**
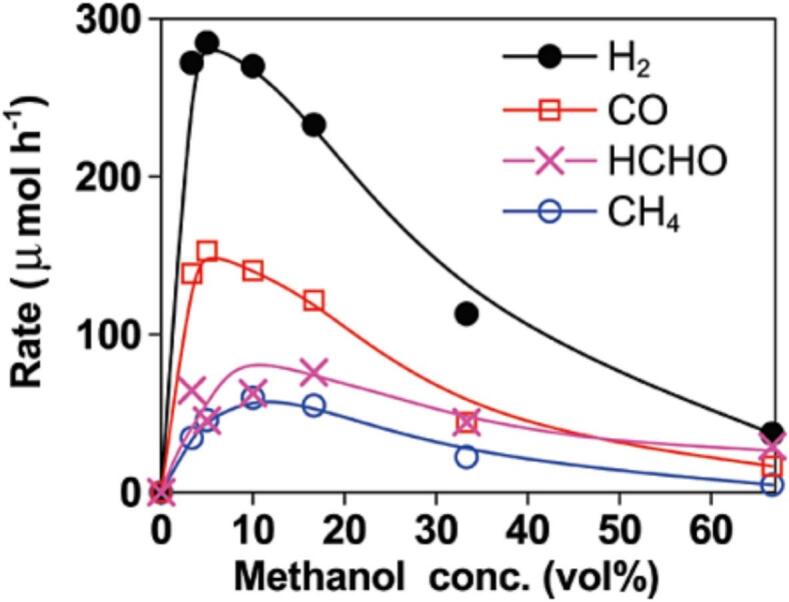
Rates of production of major products in the sonolysis of water/methanol mixtures in the presence of Au/TiO_2_ (0.5 g/L) [47].

Sonocatalysis, the interaction between ultrasonication and solid particles, is a promising technique for producing hydrogen efficiently due to its high efficiency, low cost, and nontoxic nature. However, most research focuses on the oxidation of resistant pollutants, so few studies target sonocatalytic hydrogen generation specifically. Wang et al. [47] demonstrated that using Au/TiO_2_ and TiO_2_ nanoparticles with 40 kHz (50 W) ultrasonication in an argon environment significantly enhances the rate of hydrogen generation compared to using ultrasonication alone. The rate of hydrogen generation increased by factors of 54 and 16, respectively, when Au/TiO_2_ and TiO_2_ were used. These improvements were more noticeable when 4 % (v/v) methanol was present (Fig. 7). This increase was attributed to the thermal reforming of methanol and the thermal cleavage of water molecules.

Conversely, analogous processes can be understood through the mechanisms of sonocatalytic contaminant destruction. Eren et al. [48] emphasized that TiO_2_ nanoparticles contribute to turbulent flow conditions by increasing nucleation events, improving mass transport, and enhancing chemical reactivity at the catalyst surface. In addition to an augmentation in cavitation bubbles, Tuziuti et al. [49] and Zouaghi et al. [50] stated the production of active species on the TiO_2_ surface that facilitate the generation of hydrogen peroxide and hydrogen. Other scientists ascribed the catalytic increase to the thermal energy and sonoluminescence generated during ultrasonication. This process creates holes on the TiO_2_ surface and produces hydrogen molecules and hydroxyl radicals [51,52].

Several researchers have investigated combining ultrasonication with photolytic or photocatalytic processes to increase hydrogen formation. These works suggest that further examination of the underlying processes, efficiencies, and influencing factors is necessary to improve control and optimization of these methods. For instance, Harada et al. [53] discovered that using US/photolytic and US/photocatalytic techniques reduced hydrogen output by 13.8 % and 26.68 %, respectively, when using a 200 kHz and 200 W ultrasonication setup in an argon environment, as opposed to the sole ultrasonication. Penconi et al. [10] presented an alternative to sonocatalysis, US/photocatalysis, which increased the hydrogen generation rate by 16.82 % at 38 kHz and 50 W. Other researchers have reported comparable gains using various catalysts [11], [54]. These results underscore the need for further research on these synergistic processes, as their effectiveness depends heavily on the operating parameters and employed materials.

**Fig. 8:**
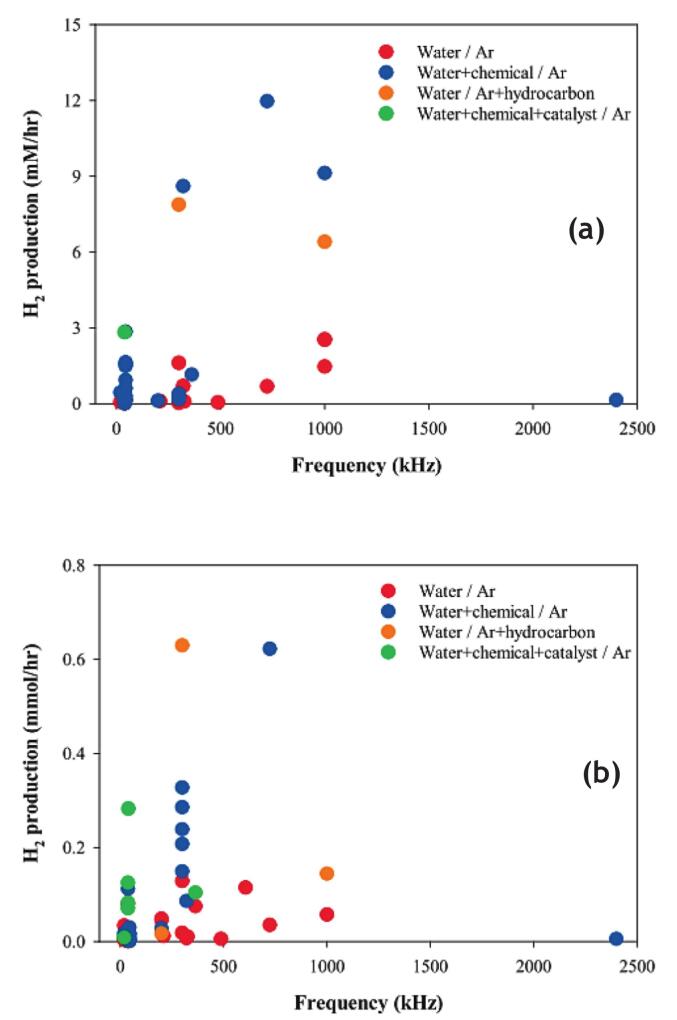
Sonolytic and US-assisted production of hydrogen from different research (regardless of the ultrasonication reactor conditions) in terms of mM/h (a) and mmol/h (b). The data (the highest value for each experimental condition) was divided into four categories: (water-Ar), (water-chemical-Ar),(water-Ar-hydrocarbon), and (water-chemical-catalyst-Ar) [12].

Recent efforts by Choi et al. [12] examined developments in ultrasonication and US/hybrid techniques for hydrogen generation, as well as how these methods could participate to completing the sustainable development goals (Table 5). The authors organized their research into four categories: water saturated with argon, a mixture of water and a liquid chemical saturated with argon gas, water saturated with a mixture of argon and hydrocarbon gases, and a mixture of water and a liquid chemical saturated with argon in the presence of a solid catalyst. Fig. 8a and 8b summarize hydrogen production data. These findings were gathered regardless of changes in the headspace volume or other ultrasonication reactor parameters. Fig. 8b shows the hydrogen production rates: water-Ar: 0.4–128.6 μmol/h, water-chemical-Ar: 0.8–622.6 μmol/h, water-Ar-hydrocarbon: 17.1–629.9 μmol/h, and water-chemical-catalyst-Ar: 8.4–282.3 μmol/h. The respective hydrogen production rates in pure water for frequency ranges of *f* < 100 kHz, 100 ≤ *f* < 1000 kHz, and *f* ≥ 1000 kHz were found to be 0.4–80.0, 5.6–128.6, and 57.3 μmol/h, respectively [12]. Including organic compounds such as methanol, ethanol, isopropanol, n-propanol, glycerol, t-butanol, dimethyl sulfoxide, ethylene glycol, and formic acid, greatly increased hydrogen production via ultrasonication [12]. The rates of hydrogen generation were 0.8–112.0 μmol/h for *f* < 100 kHz (in the presence of ethanol, methanol, formic acid, DMSO, glycerol, ethylene glycol, isopropanol), 27.9–622.6 μmol/h for 100 ≤ *f* < 1000 kHz (in the presence of methanol, t-butanol, n-butanol, n-propanol, isopropanol, ethanol), and 5.7 μmol/h for *f* ≥ 1000 kHz (in the presence of methanol). Studies comparing pure water to water containing added chemicals showed that adding compounds increased hydrogen production by a factor ranging from 1.4 to 17.8. The highest observed increase (17.8-fold) occurred with the addition of 5 % methanol at 300 kHz and 17 W.

**Table 5:** Research on ultrasonication-driven and US/hybrid Systems for Hydrogen Production (SDG: Sustainable Development Goals) [12].

The focus of the study	Key points	SDGs and targets	Ref.
US/photocatalytic generation of H_2_ using TiO_2_ in water	• Sonolytic, sonophotolytic, sonocatalytic, and sonophotocatalytic processes were compared. • The highest generation of H_2_ was obtained in sonolytic process.	• SDG-7: Ensure access to affordable, reliable, sustainable and modern energy for all. Target 7.3 • SDG-12 Ensure sustainable consumption and production patterns. Target 12.2	[53]
US/photocatalytic generation of H_2_ using rare earth catalysts in water/ethanol mixture	• Rare earth catalysts (La_0.5_Ga_0.5_InO_3_, La_0.8_Ga_0.2_InO_3_, S:La_0.8_Ga_0.2_InO_3_) were synthesized and used. • The highest generation of H_2_ was obtained in sonophotocatalytic proces.	• SDG-7: Ensure access to affordable, reliable, sustainable and modern energy for all. Target 7.3 • SDG-9: Build resilient infrastructure, promote inclusive and sustainable industrialization and foster innovation. Target 9.4 • SDG-12: Ensure sustainable consumption and production patterns. Target 12.5	[11]
Sonolytic generation of H_2_ using Au/TiO_2_ in water/alcohol mixtures	• TiO_2_ and Au/TiO_2_ were compared for the H_2_ generation under various water/alcohol mixtures. • The highest generation of H_2_ was obtained in sonocatalytic process using methanol and Au/TiO_2_.	• SDG-7: Ensure access to affordable, reliable, sustainable and modern energy for all. Target 7.3 • SDG-12: Ensure sustainable consumption and production patterns. Target 12.2	[47]
US/photocatalytic generation of H_2_ using rare earth catalysts in water/ethanol mixture	• Rare earth catalysts using La, Gd, Y, and Yb were synthesized and used. • The highest generation of H_2_ was obtained in sonophotocatalytic process using S: Y_0.8_ Ga_0.2_InO_3_.	• DG-7: Ensure access to affordable, reliable, sustainable and modern energy for all. Target 7.3 • SDG-9: Build resilient infrastructure, promote inclusive and sustainable industrialization and foster innovation. Target 9.4 • SDG-12: Ensure sustainable consumption and production patterns. Target 12.5	[10]
Sonocatalytic generation of H_2_ using metal oxides in water	• Macro and nano sized metal oxides (ThO_2_, ZrO_2_, and TiO_2_) were synthesized and used. • The highest generation of H_2_ was obtained in sonocatalytic process using TiO_2_.	• SDG-7: Ensure access to affordable, reliable, sustainable and modern energy for all. Target 7.3 • SDG-12: Ensure sustainable consumption and production patterns. Target 12.2	[57]
Sonolytic generation of H_2_ in water/alcohol mixtures	• The highest generation of H_2_ was obtained in sonolytic process using water/methanol mixture. • Additional H_2_ generation was observed after the end of ultrasonication.	• SDG-7: Ensure access to affordable, reliable, sustainable and modern energy for all. Target 7.3 • SDG-12: Ensure sustainable consumption and production patterns. Target 12.2	[62]
Sonolytic generation of H_2_ in water	• The highest H_2_ generation was obtained in sonolytic process at 20 kHz. • The sonochemical H_2_ generation was simulated under various conditions (four gases and four frequencies).	• SDG-7: Ensure access to affordable, reliable, sustainable and modern energy for all. Target 7.3 • SDG-12: Ensure sustainable consumption and production patterns. Target 12.2	[14]

The rate of hydrogen production was reported to be 2.5 to 4.9 times higher in an argon atmosphere with added hydrocarbon gases (butane, propane, ethane, methane, ethylene, and acetylene) than in an argon environment only [55], [56]. Among the examined additives, butane led to the highest rate of hydrogen generation [55]. Conversely, minimal hydrogen was produced when the ethane level exceeded 40 % [55]. Similar to the inhibitory impact observed with high alcohol concentrations in this study, these results suggest that high hydrocarbon concentrations can significantly hinder sonolytic hydrogen production.

As previously mentioned, solid catalysts, such as TiO_2_, TiO_2_/Au, ThO_2_, and rare-earth catalysts, have been studied. Using TiO_2_ at frequencies of 200 and 362 kHz did not significantly increase hydrogen generation [53], [57]. Nevertheless, notable enhancements were produced by other catalysts. For example, in a 4 % methanol solution, TiO_2_/Au increased hydrogen production by 83 times compared to the absence of a catalyst [47]. ThO_2_ increased hydrogen production threefold in water at 40 and 20 kHz, respectively, in comparison to water without a catalyst [57]. Furthermore, a study comparing sonocatalysis and US/photocatalysis revealed modest increases in hydrogen production ranging from 1.1 to 1.2 times [10], [11].

## 4 Prospective Perspectives

This section is focused on the US/electrochemical technique because it has proven to be the most promising among the tested ultrasonication-hybrid methods. However, research on US/electrochemical hydrogen generation is still in its early stages. Although preliminary research has been conducted, several critical areas require further investigation. These include the impact of various factors on the technique, such as sonoreactor design, electrolyte properties, ultrasonication frequency, ultrasonication power, electrode materials, and dimensions. The exact manner in which ultrasonication contributes to hydrogen formation remains unclear. Additionally, material, such as membranes, and electrode deterioration under ultrasonication may shorten their lifespan. The consequences of extended operation and the effect of ultrasonication on the solution's degassing process are also unclear.

Future studies should put the emphasis on combining electrocatalysis and ultrasonication to efficiently produce hydrogen on a large scale through water splitting. Moreover, more attention should be given to quantifying hydrogen generation. Though various works have addressed this topic, they only provide partial insight. Thoroughly measuring both hydrogen and oxygen is required to determine the feasibility of scaling up procedures from the laboratory stage to the pilot or industrial stage. To maximize the effects of ultrasonication, more research is needed, possibly involving multifrequency systems and the careful positioning of irradiating surfaces. In addition, systematic evaluation of experimental parameters should guide the optimization of sonoreactor design in US/electrochemical systems.

Lastly, producing hydrogen from non-aqueous liquids using ultrasonication and US/electrochemical methods is an intriguing yet largely unexplored field. Despite its potential, this topic has not received much attention in the literature.

## 5 Conclusions

Many environmentally friendly methods of producing hydrogen have been proposed as alternatives to hydrocarbon reforming. One of the most sustainable methods is water electrolysis driven by renewable energy sources. Due to its technological advantages, ultrasonication has become a worthwhile technique for enhancing the efficacy of water electrolyzer systems. Although hydrogen generation via US/electrochemistry is a relatively new technology, it has been extensively studied. Nevertheless, more thorough research is required to comprehend the effects of important factors, such as ultrasonication frequency, power, intensity, electrolyte composition, and electrode materials. To lay the groundwork for future research and development in this exciting field, this review evaluates current developments, challenges, and prospects in US/electrochemical hydrogen generation.

According to published research, the effectiveness of producing hydrogen without ultrasonication ranges from 60 % to 75 %. However, introducing ultrasonication increases the efficiency range to 80 %–85 %. Moreover, a positive correlation has been found between efficiency and electrolyte content (5 % to 18 %). Furthermore, under certain electrolyte concentrations and high current density conditions, ultrasonication has been shown to reduce energy usage by 10 % to 25 %. Although ultrasound introduces an additional energy demand, these findings suggest that the net effect can be favorable for overall efficiency when operational parameters are carefully optimized. Nevertheless, it is important to note that measuring ultrasonic efficiency remains challenging, since quantification of acoustic energy transfer depends strongly on sonoreactor design, liquid properties, and transducer-liquid coupling. Current methods, such as calorimetric measurements, provide only approximate estimations, highlighting the need for more rigorous and standardized approaches.

In addition to US/electrochemical methods, researchers have studied other US/hybrid systems to produce hydrogen. Sonocatalysis and US/photocatalysis use ultrasonication with solid catalysts or light-assisted reactions to increase hydrogen production. While some studies found improvements in hydrogen generation, others did not. Likewise, using methanol as a sacrificial agent alongside ultrasonication greatly increases hydrogen production by enhancing water molecule cleavage and thermal reforming. Researchers have also investigated US/photocatalytic methods for creating hydrogen. However, the mixed results (inhibition and augmentation) highlight the dependence of these techniques on specific materials, frequencies, and experimental conditions.

Despite impressive advancements in US/hybrid techniques, forthcoming explorations must focus on maximizing the synergy between ultrasonication and other catalytic methods. This requires a thorough evaluation of experimental settings, the development of advanced catalysts, and a deeper understanding of the processes involved. An organized approach to sonoreactor design, ultrasonication parameters, and electrode materials is indispensable for optimizing the effectiveness and sustainability of sonolytic systems. These initiatives will facilitate the way for large-scale, cost-effective hydrogen production using ultrasonication-assisted technology.

## CRediT authorship contribution statement

**Slimane Merouani:** Writing – review & editing, Writing – original draft, Visualization, Validation, Supervision, Software, Resources, Project administration, Methodology, Investigation, Formal analysis, Data curation, Conceptualization. **Aissa Dehane:** Writing – review & editing, Visualization, Validation, Software, Resources, Methodology, Investigation, Formal analysis, Data curation, Conceptualization. **Oualid Hamdaoui:** Writing – review & editing, Visualization, Validation, Supervision, Resources, Project administration, Methodology, Investigation, Funding acquisition, Formal analysis, Data curation, Conceptualization.

## Declaration of competing interest

The authors declare that they have no known competing financial interests or personal relationships that could have appeared to influence the work reported in this paper.
